# Impact of guidance issued during COVID-19 to expand take-home doses of opioid agonist treatment (OAT) in Ireland: protocol for a population-based analysis of prescribing practices and patient outcomes 2018 to 2023

**DOI:** 10.12688/hrbopenres.14044.1

**Published:** 2025-02-11

**Authors:** Gráinne Cousins, Louise Durand, Kathleen Bennett, Andy O'Hara, Des Crowley, Suzi Lyons, Eamon Keenan

**Affiliations:** 1 School of Pharmacy and Biomolecular Sciences, Royal College of Surgeons in Ireland, Dublin, Leinster, Ireland; 2 Data Science Centre, Royal College of Surgeons in Ireland, Dublin, Leinster, Ireland; 3 UISCE - National Advocacy Service for People who use Drugs in Ireland, Dublin, Ireland; 4 Irish College of General Practitioners, Dublin, Leinster, Ireland; 5 National Health Information Systems, Health Research Board, Dublin, Ireland; 6 National Social Inclusion Office, Health Service Executive, Dublin, Ireland

**Keywords:** Opioid Agonist Treatment, Take-home dosing, Opioid Use Disorder, Covid-19, retention, mortality

## Abstract

**Background:**

It is increasingly suggested that clinical guidelines and practices be updated to permanently expand relaxation around access to opioid agonist treatment (OAT) take-home doses after COVID-19. Despite a risk of OAT drug diversion, flexibility in take-home doses is valued by patients and associated with improved quality of life and retention. However, few studies have examined the effects of changes to take-home dose policies on prescribing practices and patient outcomes, with mixed results.

**Aims:**

This protocol relates to three inter-related studies. The first study will examine the impact of guidance issued on March 13th 2020 to all clinicians involved in the delivery of OAT to give the maximum number of take-home doses having given due consideration to the safety of the patient, on prescribing practices for take-home doses of methadone and buprenorphine in primary care. The second study will examine the association between increased take-home doses of OAT following March 13th 2020 guidance and treatment discontinuation in primary care. The third study will examine methadone-related deaths in Ireland before and after the guidance issue, and whether methadone-related deaths varied by whether the deceased was on OAT treatment at the time of death.

**Methods:**

Retrospective observational studies will be carried out. The first study will use a time series design to examine changes in prescribing practices of take-home doses. The second study will use a retrospective cohort study design with proportional hazard Cox models to evaluate the association between increased take-home doses and treatment discontinuation. The third study will use a repeated cross-sectional study design with interrupted time series analysis, stratified by OAT treatment status, to assess changes in methadone-related deaths.

**Discussion:**

It is anticipated that the studies will generate evidence with potential to inform both clinical and policy decision making with respect to take-home dosing of OAT.

## Introduction

Ireland has one of the highest rates of problematic opioid use in Europe, with an estimated prevalence rate of 7 per 1,000 population, corresponding to 19,875 problematic opioid users in 2019 ^1^. Drug overdose remains the primary cause of mortality among people with an opioid use disorder (OUD), and is increasing in the USA, the UK, Canada and across Europe, including Ireland ^2^. Preventive interventions, including opioid agonist treatment (OAT), contribute to reducing overdose mortality. OAT, with methadone or buprenorphine, is first line treatment for OUD, and is available free of charge to all individuals with OUD in Ireland as it is safe and effective in suppressing illicit opioid use ^3, 4^, improving mental and physical well-being ^5^, and reducing risk of all-cause, and overdose mortality ^6^. However, a recent global systematic review identified that mortality rates are six times higher when a person drops out of OAT, with the greatest risk observed in the first four weeks post treatment cessation ^7^. These findings suggest that enhancing treatment retention is critical for preventing mortality among people with OUD. Nevertheless, 6-month retention rates remain low, typically falling between 30% and 50% ^8^.

One potential challenge to treatment retention is the prolonged requirement for daily observed dosing, also referred to as supervised dosing, in community pharmacies or addiction clinics ^8^. The National Clinical Guidelines for OAT in Ireland, developed in 2016, recommend methadone as the drug of first choice in the treatment of OUD, with daily supervised consumption during the induction and stabilisation phase. In the maintenance phase, when a person demonstrates ongoing stability, a reduction from daily supervised consumption may be considered, with a maximum of 6 days’ supply of take-home doses. Although the timing and frequency of take-home doses of OAT differ between countries, patients are generally only considered eligible for progressively unsupervised (take-home) dosing after completing a minimum time in treatment with steadily negative drug screening tests ^7^. These guidelines acknowledge that facilitating access to take-home doses, with the objective of retaining a person in OAT and therefore reducing their individual risk of death needs to be balanced against the risk of increased availability of illicit methadone or buprenorphine resulting from diversion, raising the risk of methadone or buprenorphine related mortality at a population level.

The public health measures introduced in 2020 to suppress COVID-19 made OAT provision under existing regulations and clinical guidelines difficult, as services such as supervised dosing are profoundly dependent on regular in-person health care delivery ^9^. In response to these challenges, many countries introduced longer take-home dosing policies for dispensed OAT medications ^10^. Contingency OAT guidelines, recommending increased access to buprenorphine and the relaxation of take-home dosing, were introduced in Ireland in March 2020, to facilitate quick and uninterrupted access to OAT during the pandemic. On March 13 ^th^ 2020, guidance was issued to all clinicians involved in OAT delivery to give the maximum number of take-home doses having given due consideration to the safety of the patient ^11^.

It is increasingly suggested that clinical guidelines and practices should be updated to permanently expand relaxation around access to OAT take-home doses ^10, 12– 14^, as flexibility in take-home doses is perceived positively by patients and associated with improved retention and quality of life ^15– 17^. However, few studies have examined the effects of changes to take-home dose policies on prescribing practices and patient outcomes, with mixed results ^7, 14, 18– 20^. A study conducted in Ontario, Canada observed that the flexibility in take-home dosing was primarily seen in people who were already receiving take-home doses prior to the pandemic. By November 2020, prescribing for take-home dosing had largely returned to pre-pandemic patterns ^18^. Nevertheless, evidence from Canada indicates that providing increased take-home doses of OAT was linked to reduced treatment dropout at six-months, and with no increase in overdose mortality during the same period ^7^. While encouraging, these findings should be interpreted with caution as overdose deaths were only examined among those in treatment and for a relatively short duration of follow-up ^7^. Mixed evidence is emerging from the US regarding mortality ^14, 20^, with one study supporting a permanent expansion of take-home dosing as they observed no change in methadone-related deaths following increased take-home doses ^14^, and another warning against permanently relaxing take-home dosing as they observed an increase in methadone-related deaths after the policy change ^20^. The situation in Europe may be different to North America as the European Union Drugs Agency (EUDA) reports an increasing burden of diversion and misuse of OAT medications, with drug-related deaths and treatment demand associated with these medications increasing over the past decade. A retrospective study of post-mortem toxicology of OAT-related deaths in England, observed that methadone-related mortality grew by 64% in the first wave of COVID-19, and this increase occurred in cases where there was no methadone prescription at time of death, possibly from diversion arising from increased access to take-home doses. Prescribed and non-prescribed buprenorphine related mortality remained low and did not significantly change ^21^. In Ireland, methadone is the most common opioid implicated in drug poisoning deaths, with numbers increasing between 2012 and 2021 ^22^.

The aim of this study is to evaluate the impact of guidance issued during COVID-19 to expand take-home doses of methadone or buprenorphine to patients with an OUD in Ireland, on prescribing practices for OAT, treatment discontinuation and methadone-related deaths. The aim will be addressed through the three interlinked objectives:

     (1) Examine the impact of guidance issued on March 13 ^th^ 2020 to all clinicians involved in the delivery of OAT to give the maximum number of take-home doses having given due consideration to the safety of the patient, on prescribing practices for take-home doses of methadone and buprenorphine in primary care

     (2) Assess the association between the clinical decision to increase take-home doses of OAT following guidance issued on March 13 ^th^ 2020 on treatment discontinuation in primary care

     (3) Examine methadone-related deaths in Ireland before and after the national guidance issued to clinicians to expand take-home methadone doses during the COVID-19 pandemic, and whether methadone-related deaths varied by whether the deceased was in active OAT treatment at the time of death

## Methods

### Data sources

***Pharmacy claims.*** All OAT (methadone and buprenorphine) primary care prescriptions dispensed in community pharmacies in Ireland are recorded on the Health Service Executive Primary Care Reimbursement Services Opioid Substitution Treatment Scheme (PCRS–OSTS). Anonymised individual level dispensing records for methadone and buprenorphine for the years 2018 to 2023 inclusive will be provided for this project. Records include patient sex, year of birth, anonymised prescribing doctor number, geographical area, drug dispensed, prescription start and end dates, daily dose, number of days at dose, total quantity dispensed, supervised dosing in pharmacy, and number of days supervised. Drug dispensed are coded using the World Health Organisation’s Anatomical Therapeutic Chemical classification.

***Drug poisoning deaths.*** The National Drug Related Death Index (NDRDI) is an epidemiological database that records all poisoning deaths by drugs and/or alcohol. It follows the EUDA standard protocol to collect data on drug-related deaths ^23^. Drug poisoning deaths are defined as deaths directly due to the toxic effect of one or more drugs, as directed by the Coroner on the certificate of death registration and/ or the record of verdict. Up to 15 drugs implicated in drug poisoning deaths by the Coroner are included in the NDRDI. Anonymised individual level data on drug poisoning deaths will be provided for the years 2018-2023, including the deceased’s month and year of death, geographic area, socio-demographic information (year of birth, sex, homeless status at time of death), history of chronic pain, problem drug use at time of death (history of opioid dependency; history of opioid use; history of previous overdose), drug treatment history (on OAT at the time of death), and whether methadone and/or buprenorphine were implicated in the poisoning death.

### Study 1. Impact of guidance recommending increased access to take-home doses of OAT medications on OAT take-home dose prescribing in primary care

***Design.*** We will conduct an interrupted time series design of all patients dispensed methadone or buprenorphine in primary care in Ireland, as recorded in the PCRS-OSTS, between January 2018 and December 2023.

***Outcome.*** The percentage of people dispensed a range of take-home dosing categories (0, 1 to 6, 7 to 13, ≥14 days) of (a) methadone and (b) buprenorphine will be calculated for each week of the study period. The numerator will be the weekly count of people dispensed each category of take-home dosing, with the total number of people dispensed the medication during the same week as the denominator.

***Statistical analysis plan.*** Similar to a recent Canadian study ^18^, we will use autoregressive integrated moving average (ARIMA) models to examine the impact of guidance issued to prescribers on the weekly percentage of people dispensed each category of take-home dose ^24^. Any underlying long-term trend will be assessed through confirming stationarity using the augmented Dickey-Fuller test ^18, 25^. The final models will be identified using the residual autocorrelation function (ACF), partial autocorrelation function (PACF), and inverse autocorrelation function plots and the Ljung-Box test for white noise ^18, 24, 25^. To identify the impact of the guidance, a step change function will be included in the model taking the value of 0 before guidance release on March 13 ^th^ 2020 and 1 afterwards ^24^.

Analysis will be stratified by OAT drug (methadone or buprenorphine), sex and age class to identify any specific subgroup patterns. As there may be a reluctance to prescribe take-home doses to patients on high dose methadone due to a higher risk of opioid-related overdose than buprenorphine ^6^, we will also stratify by methadone dosage (maximum methadone daily dose <100 mg vs. ≥100 mg). In addition, to account for practice variation at the prescriber level, we will stratify models by prescriber OAT practice size. For each prescriber, the OAT practice size will be defined as the total number of unique patients who were prescribed methadone or buprenorphine during the 4 weeks prior to the guidance release, and categorised into quartiles.

### Study 2 – Association between clinical decision to increase number of take-home doses of OAT and OAT discontinuation in primary care

***Design.*** We will conduct a retrospective cohort study to examine the association between increased take-home dosing and treatment discontinuation among people in active treatment with OAT in Ireland on March 13 ^th^ 2020 (i.e. before the introduction of guidance to increase take-home dosing). The accrual window will be defined as the four weeks preceding the guidance release (February 15 ^th^ to March 13 ^th^ 2020). The cohort will be defined as individuals dispensed OAT through the PCRS-OSTS on at least 14 out of 28 days during the accrual window and dispensed on March 13 ^th^ 2020.

**Figure 1.**
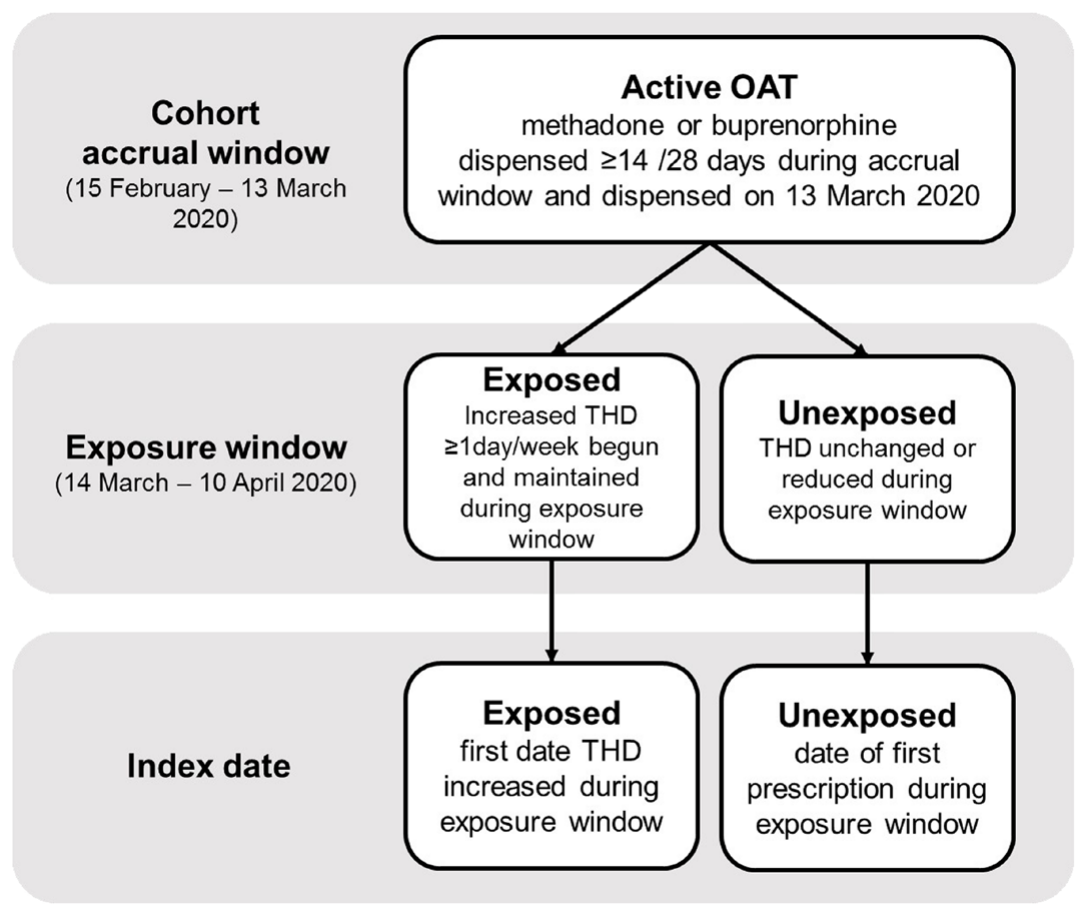
Definition of Study Cohort and Exposure Groups in Study 2.

***Exposure: increase in OAT take-home doses.*** Individual baseline take-home dosing regimen will be defined as the highest weekly number of take-home doses observed during the accrual window. The exposure window will be defined as the 4 weeks following the guidance change (March 14 ^th^, 2020, to April 10 ^th^, 2020). We will calculate the number of take-home doses on each OAT prescription during the exposure window. Using these data we will classify individuals as exposed if they experience an increase in their weekly take-home dosing by at least ≥1 day(s) during the exposure window compared to their baseline regimen. The index date will be defined as the first date of an increase in take-home dose level and to be considered exposed, this increase must be maintained or surpassed for the rest of the exposure window. Individuals whose take-home dose regimen did not meet this criteria will be classified as unexposed. The index date for the unexposed group will be defined as the date of the first prescription during the exposure window. All unexposed individuals will be required to be actively treated with methadone or buprenorphine on this assigned index date and until the end of the exposure period to ensure comparability between groups. Figure 1 displays the definition of the study cohort and exposure groups.

***Outcome.*** OAT prescription coverage will be determined for each individual using the recorded prescription details. The primary outcome will be the time to treatment discontinuation, defined as the time between index date and first subsequent OAT discontinuation. OAT discontinuation will be defined as not receiving a new methadone or buprenorphine prescription within 14 days of the end of coverage of the previously dispensed OAT prescription. We use this definition based on previous studies ^7, 8^, and following consultation with clinical and Patient and Public Involvement (PPI) partners.

***Statistical analysis plan.*** We will use a Cox proportional hazards (PH) model to compare treatment discontinuation at both 6 and 12 months between exposed and unexposed individuals, adjusting for potential confounders. Based on previous literature ^8^ and clinical judgement, potential confounders include: age, sex, age at first OAT treatment in primary care, prescriber OAT practice size (as defined above), methadone or buprenorphine daily dose, and region. Observations will be right-censored at the 6 and 12 months endpoints. The PH assumption will be confirmed by visually inspecting the log-negative-log survival curves and the Schoenfeld residuals to examine model fit. Where the PH assumption is not verified, stratified models or time interaction models will be used if appropriate. Adjusted hazard ratios and corresponding 95% Confidence Intervals (CIs) will be presented.

A sensitivity analysis will be conducted using a continuous variable for exposure as the number of additional days receiving take-home doses of methadone or buprenorphine during the exposure window compared to the baseline level. Furthermore, the proposed analysis is based on changes to patients’ take-home dose levels during the exposure window, and does not account for any additional changes in dispensing patterns over the study follow-up. Therefore, we will also model time to discontinuation accounting for variations in patients take-home doses over time by including take-home dose as a time-varying covariate using an extended Cox model ^26^.

### Study 3. Methadone-related deaths before and after prescribing guidance to expand take-home methadone doses

***Design.*** Using drug-related death data, we will conduct a repeated cross-sectional study examining the number of methadone-related deaths between 2018 and 2023. All methadone and buprenorphine related deaths (alone or in combination with other substances) from January 2018 to December 2023 recorded by the NDRDI will be included.

***Outcome.*** The primary outcome will be the bi-monthly number of methadone-related deaths, defined as deaths directly due to the toxic effect of methadone, as directed by the Coroner on the certificate of death registration and/ or the record of verdict, between January 2018 and December 2023. We will report the number of buprenorphine-related deaths but anticipate the numbers will be too low to analyse. We chose bi-monthly, as monthly figures may be too small, based on data from the NDRDI which reports 122 to 129 annual methadone-related deaths between 2018–2021 ^22^.

***Statistical analysis plan.*** Interrupted time series analysis (ITSA) will be used to model trends in methadone-related deaths. We will conduct separate segmented regression models for the primary outcome, assessing change in bi-monthly level and slope, and present regression coefficients (β) and 95% CIs before and after the guidance change. Any potential lag time between the guidance change and its implementation will be informed by the first study. To assess for residual autocorrelation, ACF and PACF plots will be visually inspected and the Ljung-Box test for white noise will be used ^24^. In light of recent evidence of an increase in methadone-related deaths among people who were not prescribed OAT at time of death in the UK in 2020 (following expansion of take-home dosing) ^21^, we will conduct a stratified analysis for (1) deaths among people prescribed OAT at time or death and (2) deaths among those not prescribed OAT.

We will also assess whether bi-monthly non-methadone poisoning deaths (i.e. drug-related deaths where methadone was not implicated by the Coroner on the certificate of death registration and/ or the record of verdict) provide a secular trend comparison, which will help assess whether a change in the trend line of methadone-related deaths is associated with the take-home policy change or could be attributed to other factors affecting trends in drug overdose deaths more generally. As suggested by Harris and colleagues, non-methadone deaths satisfy the 2 *a priori* criteria for a secular trend variable: a theory-based association between methadone and non-methadone deaths (i.e., methadone and non-methadone related deaths may be subject to the same broader social factors, including COVID-19 related context e.g. lockdowns, social distancing, self-isolation, closure of non-essential services, increase in telemedicine, and restrictions on public gatherings etc.) and the absence of a theory-based association with the guidance change (i.e., trends in non-methadone poisoning deaths are not dependent on a change in the methadone take-home policy) ^19^. A third, empirical, criterion to consider is whether methadone and non-methadone poisoning deaths are closely or moderately correlated before the policy change. If the two outcomes are not correlated, in the pre-intervention period then non-methadone poisoning deaths is not appropriate for post-intervention comparison. Two recent studies, reporting conflicting findings, used non-methadone poisoning deaths for secular trend purposes, but without providing empirical justification ^14, 20^. We will use Spearman ρ to measure the pre-intervention secular trend correlations between methadone and non-methadone poisoning deaths before the guidance change.

This project has been approved by the Royal College of Surgeons in Ireland Research Ethics Committee (REC202407028) on 10 ^th^ September 2024. The studies were designed to comply with the European General Data Protection Regulation (GDPR) 2018, the Data Protection Act 2018, and the relevant provisions of the Data Protection Act 2018 (Section 36(2)) (Health Research) Regulations.

Since only anonymised data will be used in this project, it falls outside the scope of the GDPR, and participant consent was waived by the ethical approval committee. The data controllers will satisfy themselves that the data is truly anonymous such that information related to an individual entity or person cannot be directly or indirectly identified. No information available to the researchers will allow for re-identification of individuals within the data. We will implement good data management practices and security measures for all information used in this study, including the establishment of transparent data sharing agreements with data controllers. Furthermore, we will ensure that data is published only in aggregated formats, with any data point representing fewer than five individuals being suppressed to maintain confidentiality. Comprehensive data protection impact assessments have been conducted in compliance with the Data Protection Act. Data security and management strategies will focus on ensuring data quality, and using encrypted, password-protected storage devices accessible only to authorised researchers.

All analyses will be conducted using SAS software (Enterprise Guide v 7.1, Base v 9.4; SAS Institute, Cary, NC) and use a type-1 error rate of 0.05. We will present our findings following the guidelines outlined in the Reporting of Studies Conducted Using Observational Routinely Collected Health Data statement ^27, 28^.

## Discussion

### Strengths and limitations

The changes in guidelines implemented during the COVID-19 pandemic demonstrated the healthcare system's capacity for rapid and substantial adaptation. However, there is a need for published evidence on prescribing practices and patient outcomes associated with these changes ^10^. This study aims to address significant gaps in knowledge by investigating the impact of guidance on take-home dosing in primary care in Ireland, as well as key outcomes such as discontinuation and mortality. By including all patients on a national prescribing register over a seven-year study period, the external validity will be high. We will use appropriate statistical methods such as ARIMA and PH models and conduct gender and age-sensitive analyses to provide a comprehensive perspective. To the authors’ knowledge, these will be the first studies to report on the impact of COVID-19 related OAT guidance on observed prescribing practices, treatment discontinuation and OAT drug related mortality in Ireland. In a context of mixed international findings, this will provide important evidence to inform future service delivery.

However, some limitations of the studies can be anticipated. Firstly, the observations will be limited to changes in take-home doses and discontinuation in primary care settings, excluding data from specialist centres typically attended by less stable or homeless patients. Approximately 40% of OAT patients in Ireland are treated in primary care settings ^29^. For treatment discontinuation outcomes, interruption of community dispensing for 14 days or more will be classified as treatment cessation. However, patients may experience interruptions due to transfer to specialist services, hospital, prison, moving abroad, or death, which will be incorrectly classified as discontinuing treatment. There will be no possibility of quantifying this misclassification bias, however the primary care OAT cohort is generally regarded as more stable, and less likely to be incarcerated, or hospitalised for overdose than people attending specialist clinics. Secondly, for the methadone-related mortality, although we can determine whether the deceased was on OAT at the time of death, we will be unable to ascertain whether methadone was prescribed or diverted, as there is no linkage between prescribing practices and mortality data. Thirdly, the simultaneous increase in individuals receiving OAT in Ireland ^30^, and additional OAT changes such as reduced frequency of urine drug testing ^16^, alongside the implementation of guidance on take-home dosing introduce residual confounding that may influence the outcomes examined. Establishing causality between the intervention and outcomes is challenging, but the study plans to use robust methods such as ITSA and comparative analysis of trends in non-methadone related deaths to address this issue. The Bradford Hill criteria framework can be used to assess causal inference, however associations may remain hypothesis generating only.

### Public and Patient Involvement (PPI)

This protocol was developed in active partnership with PPI co-author AOH, Community Coordinator at UISCE – the National Advocacy Service for People who use Drugs (PWUD) in Ireland. UISCE is currently the advocate/representative for the community of PWUD at several treatment and harm-reduction strategic committees in Ireland. Ongoing engagement with PWUD will take place, particularly those who may access OAT, throughout the lifetime of this project. Several meetings with service users (men and women) will be organised to support the development of the project, interpretation of the results and to guide meaningful dissemination among service users, ensuring this project is truly participatory from a PPI perspective. Throughout the course of this project we will adopt the Guidance for Reporting Involvement of Patients and the Public (GRIPP) standardised reporting guideline. Its use will ensure the contribution of the PPI project team member will be fully communicated in dissemination and provide evidence of the value of stakeholder, public and patient involvement in health services research.

## Ethics and consent statement

This project has been approved by the Royal College of Surgeons in Ireland Research Ethics Committee (REC202407028) on 10 ^th^ September 2024. The studies were designed to comply with the European General Data Protection Regulation (GDPR) 2018, the Data Protection Act 2018, and the relevant provisions of the Data Protection Act 2018 (Section 36(2)) (Health Research) Regulations. Since only anonymised data will be used in this project, it falls outside the scope of the GDPR. and participant consent was waived by the ethical approval committee

## Dissemination

Findings will be disseminated through publication in peer-reviewed journals and to relevant national and international conferences. We will also publish our research findings in *UISCE magazine*, a peer-led publication disseminated nationally to services attended by PWUD.

## Conclusion

There is a need for published evidence on the impact of OAT guidance changes, particularly around take-home dosing. Across three studies, this project will use routinely collected data to provide insight on changes in prescribing practices, treatment discontinuation and OAT drug-related mortality associated with the take-home dosing guidance changes introduced during the pandemic. In a context of mixed international findings, these studies have the potential to inform future policy and service delivery, benefiting patients.

## Abbreviations

ACF                         Autocorrelation Function

ARIMA                   Auto Regressive Integrated Moving Average

CI                            Confidence Interval

EUDA                     European Union Drugs Agency

GDPR                     General Data Protection Regulation

GRIPP                     Guidance for Reporting Involvement of Patients and the Public

ITSA                       Interrupted Time Series Analysis

NDRDI                   National Drug Related Death Index

OAT                        Opioid Agonist Treatment

OUD                       Opioid Use Disorder

PACF                      Partial Autocorrelation Function

PCRS-OSTS           Primary Care Reimbursement Services - Opioid Substitution Treatment Scheme

PH                           Proportional Hazards

PPI                          Patient and Public Involvement

PWUD                    People Who Use Drugs

## Data Availability

No data are associated with this article.
