# Supplementary material for: Impact of guidance issued during COVID-19 to expand take-home doses of opioid agonist treatment (OAT) in Ireland: protocol for a population-based analysis of prescribing practices and patient outcomes 2018 to 2023
Source: HRB Open Res. 2026 Mar 10;8:32. Originally published 2025 Feb 11. [Version 3] doi: 10.12688/hrbopenres.14044.3 (PMC12120409; doi:10.12688/hrbopenres.14044.3)
Supplement: Peer review history

# Reviewer report: Reviewer response for version 3

**DOI:** 10.21956/hrbopenres.15835.r54071

**Published:** 2026-03-13

**Authors:** Kristen A Morin

**Affiliations:** 1 Northern Ontario School of Medicine University, Sudbury, Ontario, Canada

Thank you. The authors have addressed all my comments.

Is the study design appropriate for the research question?

Yes

Is the rationale for, and objectives of, the study clearly described?

Yes

Are sufficient details of the methods provided to allow replication by others?

Partly

Are the datasets clearly presented in a useable and accessible format?

Not applicable

Reviewer Expertise:

Opioid agonist treament (OAT), epidemiology, health services research

I confirm that I have read this submission and believe that I have an appropriate level of expertise to confirm that it is of an acceptable scientific standard.

# Reviewer report: Reviewer response for version 2

**DOI:** 10.21956/hrbopenres.15533.r46663

**Published:** 2025-05-30

**Authors:** Eugenia Oviedo-Joekes

**Affiliations:** 1 The University of British Columbia, Vancouver, British Columbia, Canada

The authors have done a thoughtful work in this second version. I have no other comments.

Is the study design appropriate for the research question?

Yes

Is the rationale for, and objectives of, the study clearly described?

Partly

Are sufficient details of the methods provided to allow replication by others?

Partly

Are the datasets clearly presented in a useable and accessible format?

Yes

Reviewer Expertise:

Person-Centered Care in Addiction and Public Health

I confirm that I have read this submission and believe that I have an appropriate level of expertise to confirm that it is of an acceptable scientific standard.

# Reviewer report: Reviewer response for version 2

**DOI:** 10.21956/hrbopenres.15533.r46753

**Published:** 2025-05-28

**Authors:** Roman Ivasiy

**Affiliations:** 1 Yale School of Medicine, New Haven, USA

Thank you for the opportunity to review this important and timely study, which has the potential to significantly impact OAT delivery policy in Europe and globally.

 I believe there are a few recommendations that could improve the methodology, particularly in the second study of the manuscript. From my understanding, the authors are considering only the first treatment episodes of OAT for the selected cohort, which may be appropriate if most patients had only one within the observation period, but it usually does not fully reflect the real-life experiences of people who use drugs. I suggest using frailty survival models to better account for multiple treatment episodes. These models would allow for the inclusion of repeated treatment episodes.

 Additionally, I am concerned that including time-dependent covariates only in the sensitivity analysis may not be sufficient. It would be much more convincing if time-dependent covariates, such as dose and take-home dosing, were incorporated into the main model. Both of these factors have the potential to increase over time and are inevitably associated with treatment retention, as both are proven to be associated with longer retention. As the duration of OAT episodes increases, the chances of dose adjustments and extended supply days rise, making them crucial factors to include in the main analysis to keep it unbiased systematically. Therefore, controlling for the dose changes over time is essential to assess the effect of the THD.

 Additionally, I believe including some more recent studies on the topic of THD expansion during COVID in the introduction would provide readers with valuable context. Some of these studies include:

1. Ivasiy R, Madden LM, Meteliuk A, Machavariani E, et al. (2024) - ( **Ref 1**)
2. Krawczyk N, et al. (2023) - ( **Ref 2)**

Is the study design appropriate for the research question?

Partly

Is the rationale for, and objectives of, the study clearly described?

Yes

Are sufficient details of the methods provided to allow replication by others?

Yes

Are the datasets clearly presented in a useable and accessible format?

Yes

Reviewer Expertise:

Opiod Use Disorder, HIV, HCV, Healthcare Service Reserch

I confirm that I have read this submission and believe that I have an appropriate level of expertise to confirm that it is of an acceptable scientific standard, however I have significant reservations, as outlined above.

## References

[1] : The impact of emergency guidance to the COVID-19 pandemic on treatment entry, retention and mortality among patients on methadone in Ukraine. *Addiction* .2024;119(9) : 10.1111/add.16565 1585-1596 10.1111/add.16565 38807448

[2] : Synthesising evidence of the effects of COVID-19 regulatory changes on methadone treatment for opioid use disorder: implications for policy. *Lancet Public Health* .2023;8(3) : 10.1016/S2468-2667(23)00023-3 e238-e246 10.1016/S2468-2667(23)00023-3 36841564 PMC9949855

# Reviewer report: Reviewer response for version 2

**DOI:** 10.21956/hrbopenres.15533.r46662

**Published:** 2025-04-16

**Authors:** Kristen A Morin

**Affiliations:** 1 Northern Ontario School of Medicine University, Sudbury, Ontario, Canada

Thank you to the authors for their thoughtful revisions. I have no further comments

Is the study design appropriate for the research question?

Yes

Is the rationale for, and objectives of, the study clearly described?

Yes

Are sufficient details of the methods provided to allow replication by others?

Partly

Are the datasets clearly presented in a useable and accessible format?

Not applicable

Reviewer Expertise:

Opioid agonist treament (OAT), epidemiology, health services research

I confirm that I have read this submission and believe that I have an appropriate level of expertise to confirm that it is of an acceptable scientific standard.

# Reviewer report: Reviewer response for version 1

**DOI:** 10.21956/hrbopenres.15418.r46017

**Published:** 2025-03-12

**Authors:** Eugenia Oviedo-Joekes

**Affiliations:** 1 The University of British Columbia, Vancouver, British Columbia, Canada

STUDY PROTOCOL

 Impact of guidance issued during COVID-19 to expand take-home doses of opioid agonist treatment (OAT) in Ireland: protocol for a population-based analysis of prescribing practices and patient outcomes 2018 to 2023

 This protocol will examine the impact of Ireland’s COVID-19 policy change that expanded take-home opioid agonist treatment (OAT) doses. It will assess how this change influenced prescribing practices, treatment discontinuation rates, and methadone-related deaths. The protocol employs retrospective observational designs, with three studies: Study 1 analyzes changes in prescribing practices using a time series design, Study 2 assesses the association between increased take-home doses and treatment discontinuation through a cohort study, and Study 3 examines methadone-related deaths before and after the policy change using an interrupted time series analysis.

 The protocol has several strengths. The work is very timely, as take-home practices are a right that people in OAT have struggled to reclaim, and a central principle of person-centered addiction care: autonomy. The COVID-19 pandemic allows many clients to benefit from take-home dosing, however in many regions these policies are being rolled back without evidence. The present study will add substantial evidence to the current landscape of the role of policy changes regarding take-homes and clients’ outcomes. Another strength, this protocol is being developed in collaboration with people with lived/ing experience, who will partner in all aspects of the study. While the data used in the study might not be person-centered, this collaboration ensures the process takes into consideration the perspectives of those most affected by the policies and their consequences. Finally, the authors have developed three complementary studies using data from a national prescribing register over a seven-year study period that will allow for a comprehensive picture given the available data.

 The present submission could be improved in clarity in the methods, some definitions read ambiguous.  Particular attention to the subtext when writing is encouraged, to avoid the reader to make stigmatizing assumptions. While is true that regarding ‘hard outcomes’ more evidence is welcomed, there is plenty of evidence, including systematic reviews, of the benefits of take-home dosing, from the clients’ perspectives, providers perspectives, there are studies showing no association with treatment outcomes. The background gives the impression that overall, the results are mixed. This can be improved.

 Specific comments

 Background

 “Nevertheless, 6-month retention rates remain low, typically falling between 30% and 50%” This refers to Ireland? Is this common across the countries and continents mentioned? Is this for methadone and buprenorphine?

 Why do you consider “the prolonged requirement for daily observed dosing” a ‘potential challenge’ and not directly a ‘challenge’? Is there not enough evidence that shows this is a barrier to access care?

 Were the National Clinical Guidelines for OAT in Ireland (2016) developed with consultation with people of lived/living experience? Despite the guidelines, is there any data on how many clients actually benefitted from take-homes?

 The change in the guidelines in 2020 towards providing the maximum number of take-home doses does not seem to be in consideration to the safety of the patient, but in the safety of the community. These changes were to protect the community from COVID. The systems of control and stigma toward people with OUD became a secondary issue. I think it is very important to be reflective of these issues among structurally disadvantaged populations, and not continue perpetuating structural violence. This is extremely relevant since many of the incredible advances in addiction and public health gained during the COVID pandemic are being rolled back, despite the evidence (i.e., non-events) now that the system does not have to protect the community anymore with those measures.

 The authors cite the study finding “A retrospective study of post-mortem toxicology of OAT-related deaths in England, observed that methadone-related mortality grew by 64% in the first wave of COVID-19, and this increase occurred in cases where there was no methadone prescription at time of death […]” concluding that this increase was “possibly from diversion arising from increased access to take-home doses”. This is a simplification of the study of Aldabergenov et al. First, they cite a long list of protecting factors of those with a prescription. Then, they provide evidence from other studies that take-home diversion has not been proved to drive negative outcomes, beyond specific cases. What is the evidence the authors have to indicate that a 64% increase in methadone mortality is due to take-home diversion? How are these calculations done? Given that the methadone levels detected were unchanged in both groups relative to previous years, and the prescribed group had higher levels than the non-prescribed (although not significant), how does that explain ‘sharing’ or ‘selling’ their own medication (what will they use instead?)?.

 The language from the three interlinked objective, where does it come from? In objective 1, is it necessary to include all that language that seem to come from the guidance document?

 In objective 2, You are looking at the association of the difference in take-home prescriptions before/after March 13th 2020 with treatment discontinuation? If so, this needs to be expressed in a clearer manner.

 Objective 3, “[…] and whether methadone-related deaths varied by whether the deceased was in active OAT treatment at the time of death.” This line does not read well. You mean to compare methadone-related deaths with and without an active methadone OAT prescription? (if it can be distinguished of methadone prescribed for pain)

 Methods

 For study 1, have the authors considered geographic area as a stratification? Yes, no? Why? Centralized areas and decentralized areas or urban/rural areas, for example, will present distinct patters of acceptance/need of the guidance.

 Study 2, we are talking about prescriptions increases, correct? The wording “increase TH doses” reads like the dose itself will be increased.

 The exposure variable has 2 levels: 1) maintained increase of at least one day of take-home dispensation, and 2) not meeting the prior condition. “All unexposed individuals will be required to be actively treated with methadone or buprenorphine on this assigned index date and until the end of the exposure period to ensure comparability between groups”. Isn’t time to discontinuation the outcome variable of study 2? “The primary outcome will be the time to treatment discontinuation, defined as the time between index date and first subsequent OAT discontinuation”

 Figure 1 is not self-explanatory, and it does not really help discern between the cohort and exposure groups.

 Study 3 outcome, can you clarify the definition of cause of death? Is it a code? There is some ambiguity, when one side it says “directly due to the toxic effect of methadone”, but in the other side is says “methadone-related”. Also, when more than one substance is present, how is the death coded? Does the death certificate have that certainty, or the death is attributed to the ‘toxic effect of methadone’?

 In the statistical analysis of study 3, the authors mention again that the increase in methadone-related deaths after the expansion in take-home dispensations, implying causation, citing Aldabergenov et al., offering no evidence of such connection. This is particularly relevant when the authors indicate that there is no linkage between prescribing practices and mortality data. Therefore, it is important to exercise caution particularly when writing about structurally disadvantage populations to avoid re-stigmatizing them with between the lines’ assumptions.

 Discussion (cont.)

 The authors, also in the discussion, imply that they can offset the challenge to establish causation with their robust methodology. This sentence needs to be corrected. The present study design does not allow proving causal relationships.

 Dissemination

 Regarding the dissemination in the UISCE magazine, can the authors elaborate a bit on this? Will this publication be led or co-led with people with lived/ing experience?

Is the study design appropriate for the research question?

Yes

Is the rationale for, and objectives of, the study clearly described?

Partly

Are sufficient details of the methods provided to allow replication by others?

Partly

Are the datasets clearly presented in a useable and accessible format?

Yes

Reviewer Expertise:

Person-Centered Care in Addiction and Public Health

I confirm that I have read this submission and believe that I have an appropriate level of expertise to confirm that it is of an acceptable scientific standard, however I have significant reservations, as outlined above.

# Response

**Published:** 2025-04-04

**Authors:** Louise Durand

**Affiliations:** Royal College of Surgeons in Ireland, Ireland

The present submission could be improved in clarity in the methods, some definitions read ambiguous.  Particular attention to the subtext when writing is encouraged, to avoid the reader to make stigmatizing assumptions. While is true that regarding ‘hard outcomes’ more evidence is welcomed, there is plenty of evidence, including systematic reviews, of the benefits of take-home dosing, from the clients’ perspectives, providers perspectives, there are studies showing no association with treatment outcomes. The background gives the impression that overall, the results are mixed. This can be improved.

 Specific comments

  

1. Background: “Nevertheless, 6-month retention rates remain low, typically falling between 30% and 50%” This refers to Ireland? Is this common across the countries and continents mentioned? Is this for methadone and buprenorphine?

*Response:*

 To clarify, we have modified this statement using evidence from a systematic review.

*“Nevertheless, retention remains low internationally, with a median 12-month retention rate of 57% found in a systematic review of 37 studies* *^8^* *.”*

  

1. Why do you consider “the prolonged requirement for daily observed dosing” a ‘potential challenge’ and not directly a ‘challenge’? Is there not enough evidence that shows this is a barrier to access care?

*Response:*

 We have removed “potential” and modified the manuscript accordingly.

*“One challenge to treatment retention is the prolonged requirement for daily observed dosing, also referred to as supervised dosing, in community pharmacies or addiction clinics.”*

1. Were the National Clinical Guidelines for OAT in Ireland (2016) developed with consultation with people of lived/living experience? Despite the guidelines, is there any data on how many clients actually benefitted from take-homes?

*Response:*

 Yes, the 2016 National Clinical Guidelines for OAT were developed in consultation with people with lived and living experience. A working group comprising representatives from the College of Psychiatrists of Ireland, the Irish College of General Practitioners, the Pharmaceutical Society of Ireland, and HSE Addiction Services consulted with key stakeholders including 2 service user representative groups, UISCE (Advocacy Group for People who use drugs in Ireland) and SURF (Service User Representative Forum) and staff working in the services. Expert opinion from Professor Michael Farrell, Director of the National Drug and Alcohol Research Centre at the University of New South Wales, was obtained both during the process and on completion.

 With respect to data on take-home doses, there is no published evidence regarding how many clients access/benefitted from take-home doses in Ireland over the study period.

  

1. The change in the guidelines in 2020 towards providing the maximum number of take-home doses does not seem to be in consideration to the safety of the patient, but in the safety of the community. These changes were to protect the community from COVID. The systems of control and stigma toward people with OUD became a secondary issue. I think it is very important to be reflective of these issues among structurally disadvantaged populations, and not continue perpetuating structural violence. This is extremely relevant since many of the incredible advances in addiction and public health gained during the COVID pandemic are being rolled back, despite the evidence (i.e., non-events) now that the system does not have to protect the community anymore with those measures.

*Response:*

 Thank you for this considered reflection. We totally agree with Reviewer 2 that people who use drugs are subjected to structural stigma, which is harmful to their health and wellbeing. In fact, in recent years UISCE have co-produced / co-delivered an annual workshop with co-authors for students in the RCSI on reducing stigma for people who use drugs in the health services. In our discussions for this study, our agreed aim was to understand what changes took place in Ireland following changes in guidelines, and whether those changes were sustained over time. Reviewer 2 noted that “public health gained during the COVID pandemic are being rolled back”, we do not have evidence of this in the Irish context, it is for this very reason we are undertaking this study. Furthermore, given the high levels of drug poisoning deaths in Ireland, we also wanted to understand the impact of any changes on treatment discontinuation (a known risk factor for mortality) and methadone -related deaths. This goal in no way seeks to further stigmatise people who use drugs – our goal is to accumulate evidence scientifically to inform services and generate further research questions to improve care and reduce mortality among people who use drugs in Ireland. We appreciate and acknowledge that observational epidemiological studies cannot infer causation and we are explicit that our findings are hypothesis generating (see response #15).

  

1. The authors cite the study finding “A retrospective study of post-mortem toxicology of OAT-related deaths in England, observed that methadone-related mortality grew by 64% in the first wave of COVID-19, and this increase occurred in cases where there was no methadone prescription at time of death […]” concluding that this increase was “possibly from diversion arising from increased access to take-home doses”. This is a simplification of the study of Aldabergenov et al. First, they cite a long list of protecting factors of those with a prescription. Then, they provide evidence from other studies that take-home diversion has not been proved to drive negative outcomes, beyond specific cases. What is the evidence the authors have to indicate that a 64% increase in methadone mortality is due to take-home diversion? How are these calculations done? Given that the methadone levels detected were unchanged in both groups relative to previous years, and the prescribed group had higher levels than the non-prescribed (although not significant), how does that explain ‘sharing’ or ‘selling’ their own medication (what will they use instead?)?.

*Response:*

 Thank you for your comments with respect to our citation of Aldabergenov et al. In response to “What is the evidence the authors have to indicate that a 64% increase in methadone mortality is due to take-home diversion”, we do not state that this increase is due solely to diversion – we indicate that diversion is one possibility. However, Reviewer 2 is correct we did not provide alternative explanations as put forward by the authors. We have addressed this limitation, and highlighted other possible contributions as suggested:

*“A retrospective study of post-mortem toxicology of OAT-related deaths in England, observed that methadone-related mortality grew by 64% in the first wave of COVID-19, and this increase was greatest among cases where there was no methadone prescription at time of death. The authors acknowledge that multiple factors could account for the increase in methadone-related deaths in those not prescribed OAT, including reduced access to psychological supports, harm reduction and out – reach services such as naloxone among those not in treatment. The increase in methadone-related death seen in people not prescribed it raises the possibility that an important change to the drug market that occurred during the COVID-19 pandemic in England was an increased availability of methadone. This possibility raises the question of diversion* *(Aldabergenov et al., 2022)* *.* *Prescribed and non-prescribed buprenorphine related mortality remained low and did not significantly change *(Aldabergenov et al., 2022) *. In Ireland, methadone is the most common opioid implicated in drug poisoning deaths, with numbers increasing between 2012 and 2021* (Kelleher et al., 2021). *“*

  

1. The language from the three interlinked objective, where does it come from? In objective 1, is it necessary to include all that language that seem to come from the guidance document?

*Response:*

 We included detail with respect to time, person and the specific guidance issued to ensure clarity in the study objective. We felt this was a useful means of signposting, which is reflected in the methods. However, we appreciate your comment that perhaps this level of detail is not necessary in the objective and have simplified to:

1. *Examine the impact of changes in guidance for the provision of OAT take-home doses in Ireland, on prescribing practices for take-home doses of methadone and buprenorphine in primary care*

 

1. In objective 2, You are looking at the association of the difference in take-home prescriptions before/after March 13th 2020 with treatment discontinuation? If so, this needs to be expressed in a clearer manner.

*Response:*

 Apologies for the lack of clarity with respect to objective 2. We have clarified here, also reflecting on your comments with respect to our language used in objective 1.

1. *Assess the association between increased take-home doses of OAT, following changes in guidance, and treatment discontinuation in primary care*

 

1. Objective 3, “[…] and whether methadone-related deaths varied by whether the deceased was in active OAT treatment at the time of death.” This line does not read well. You mean to compare methadone-related deaths with and without an active methadone OAT prescription? (if it can be distinguished of methadone prescribed for pain)

*Response:*

 As stated in the methods section, data on drug poisoning deaths are available from the National Drug Related Death Index (NDRDI). We have provided additional detail on this epidemiological database (see below), which clarifies the sources of data used by the NDRDI. The NDRDI determines whether a person is on OAT at the time of death through data from the national treatment register for OAT, the central treatment list. Treatment status is not in this instance determined by whether a person was in receipt of a prescription from dispensing records, so pain medications are not a confounder. To ensure further clarity, we have also provided detail on the treatment register in the ‘Setting’ section at the beginning of the methods section, which we hope clarifies the Irish system further.

*“ **Setting***

*Methadone and buprenorphine are available free of charge to all persons undergoing OAT for opioid use disorder in Ireland. In 1998 the Misuse of Drugs (Supervision of Prescription and Supply of Methadone) Regulations were introduced in Ireland, which involved the establishment of a national register, the Central Treatment List (CTL). The Misuse of Drugs Regulations were updated in 2017 to authorise access to buprenorphine or buprenorphine/naloxone for OAT on the same statutory basis as methadone. All individuals in receipt of OAT are registered on the CTL, with each person linked to one specific prescriber and a single pharmacy dispensing site. A total of 10,251 people were in receipt of OAT in 2019 *(Durand et al., 2023) *. OAT is provided in specialist outpatient addiction clinics or primary care settings, with approximately 60% of people in treatment in specialist addiction clinics *(Delargy et al., 2019; Durand et al., 2021) *. Previous studies of OAT in Ireland suggest that access to take-home doses is greater in primary care”*

***“*** ***Drug poisoning deaths.*** *The National Drug Related Death Index (NDRDI) is an epidemiological database that records all poisoning deaths by drugs and/or alcohol. It follows the EUDA standard protocol to collect data on drug-related deaths* *(EMCDDA, 2012)* *. To ensure completeness, mortality data are collected from multiple sources and cross-checked to avoid duplication. Coronial files are the primary source and include post mortem toxicology reports. Other data sources include: General Mortality Register through the Central Statistics Office (CSO), acute hospitals data via the HSE Hospital In-Patient Enquiry (HIPE) system and the CTL. Drug poisoning deaths are defined as deaths directly due to the toxic effect of one or more drugs, as directed by the Coroner on the certificate of death registration and/ or the record of verdict. Up to 15 drugs implicated in drug poisoning deaths by the Coroner are included in the NDRDI. Anonymised individual level data on drug poisoning deaths will be provided for the years 2018-2023, including the deceased’s month and year of death, geographic area, socio-demographic information (year of birth, sex, homeless status at time of death), history of chronic pain, problem drug use at time of death (history of opioid dependency; history of opioid use; history of previous overdose), drug treatment history (on OAT at the time of death as recorded in Central Treatment List), and whether methadone and/or buprenorphine were implicated in the poisoning death.”*

 We have also revised objective 3 as recommended:

  

1. *Examine methadone-related deaths before and after changes in guidance for the provision of OAT take-home doses and* *by treatment status at time of death (i.e.* *whether* *the deceased was in active OAT treatment vs. out of OAT treatment at the time of death)*

 

1. Methods: For study 1, have the authors considered geographic area as a stratification? Yes, no? Why? Centralized areas and decentralized areas or urban/rural areas, for example, will present distinct patters of acceptance/need of the guidance.

*Response:*

 Thank you for raising this excellent point. We have indeed considered stratifying/adjusting for geographic information. This is important as COVID-19 measures may have affected urban/rural territories differently, notably in term of travel restrictions. While estimated rates of problematic opioid use are 4 times higher in county Dublin compared to rest of the country (Hanrahan et al., 2022), access to OAT can be problematic in rural areas (Delargy et al., 2019) and the large majority of services are often located in Dublin. The data provided by the PCRS will include the dispensing pharmacy health region, which will give an indication of the area where people are dispensed OAT. Unfortunately this level of granularity will not allow to distinguish urban vs rural areas as some health regions include both major cities and rural areas. We anticipate being able to meaningfully separate the greater Dublin area vs the rest of the country which is the approach taken in other publications (Hanrahan et al., 2022). We included this level of stratification explicitly in the statistical plan for objective 1.

*“The analysis will be stratified by OAT drug (methadone or buprenorphine), sex, age class, and geographic area to identify any specific subgroup patterns.*”

  

1. Study 2, we are talking about prescriptions increases, correct? The wording “increase TH doses” reads like the dose itself will be increased.

*Response:*

 Reviewer 2 is correct, the exposure variable is increased number of take-home doses, not the drug dosage. We have clarified this in the text as highlighted below.

*“ Using these data we will classify individuals as exposed if they experience an increase in their weekly number of take-home doses by at least ≥1 day(s) during the exposure window compared to their baseline regimen.”*

  

1. The exposure variable has 2 levels: 1) maintained increase of at least one day of take-home dispensation, and 2) not meeting the prior condition. “All unexposed individuals will be required to be actively treated with methadone or buprenorphine on this assigned index date and until the end of the exposure period to ensure comparability between groups”. Isn’t time to discontinuation the outcome variable of study 2? “The primary outcome will be the time to treatment discontinuation, defined as the time between index date and first subsequent OAT discontinuation”

*Response:*

 Due to the design of the study we set an exposure window where all individuals need to be observed in order for us to define their exposure group, similar to existing literature (Gomes et al., 2022). As a consequence, to avoid “immortal bias”, individuals in the unexposed group need to stay in treatment at least until the end of the exposure window. We have modified the index date to take place at the end of the exposure window for both exposed and unexposed groups so individuals are at risk of discontinuation from the onset of observation period. Time to discontinuation is therefore defined as the time between the index date and first subsequent OAT discontinuation, and can only be observed after the end of the exposure window.

*“ **Exposure**: increase in OAT take-home doses. Individual baseline take-home dosing regimen will be defined as the highest weekly number of take-home doses observed during the accrual window. The exposure window will be defined as the 4 weeks following the guidance change (March 14th, 2020, to April 10th, 2020). We will calculate the number of take-home doses on each OAT prescription during the exposure window. Using these data we will classify individuals as exposed if they experience an increase in their weekly number of take-home doses by at least ≥1 day(s) during the exposure window compared to their baseline regimen. Individuals whose take-home dose regimen did not meet this criteria will be classified as unexposed. The index date will be defined as the first day after the exposure window, i.e. April 11th 2020. All unexposed individuals will be required to be actively treated with methadone or buprenorphine until the end of the exposure period to ensure comparability between groups. Patients who had their OAT drug changed (from methadone to buprenorphine or vice versa) during the accrual or exposure windows will be excluded.* *Figure 1* *displays the definition of the study cohort and exposure groups.”*

  

1. Figure 1 is not self-explanatory, and it does not really help discern between the cohort and exposure groups.

*Response:*

 Thank you for this feedback, we have taken time to reflect on your comments and have modified figure 1 to provide greater clarity on the cohort and exposure groups. We hope this is helpful to the reader.

https://hrbopenresearch.s3.eu-west-1.amazonaws.com/linked/200121.14044-Louise_Durand-Response.pdf

  

1. Study 3 outcome, can you clarify the definition of cause of death? Is it a code? There is some ambiguity, when one side it says “directly due to the toxic effect of methadone”, but in the other side is says “methadone-related”. Also, when more than one substance is present, how is the death coded? Does the death certificate have that certainty, or the death is attributed to the ‘toxic effect of methadone’?

*Response*

 Thank you for this comment. We use methadone-related death throughout the manuscript, defining it as death directly due to the toxic effect of methadone. In polydrug poisoning deaths, all drugs recorded on the death certificate have equal weight in relation to the cause of death i.e. there is not one main drug and then additional drugs. The drugs implicated on the death certificate are transcribed verbatim to the NDRDI database and then assigned ICD codes, unique NDRDI codes and ATC codes (where applicable) by NDRDI researchers. Up to 15 drugs implicated in drug poisoning deaths by the Coroner are included in the NDRDI. If methadone is deemed implicated we consider it a methadone-related death, even if other substances are also present/implicated, consistent with standard reporting practices for the NDRDI. We added a clarification to the definition of the outcome:

 “ ***Outcome.*** *The primary outcome will be the bi-monthly number of methadone-related deaths, defined as deaths directly due to the toxic effect of methadone, alone or in combination with other substances, as directed by the Coroner on the certificate of death registration and/ or the record of verdict, between January 2018 and December 2023.”*

  

1. In the statistical analysis of study 3, the authors mention again that the increase in methadone-related deaths after the expansion in take-home dispensations, implying causation, citing Aldabergenov et al., offering no evidence of such connection. This is particularly relevant when the authors indicate that there is no linkage between prescribing practices and mortality data. Therefore, it is important to exercise caution particularly when writing about structurally disadvantage populations to avoid re-stigmatizing them with between the lines’ assumptions.

*Response:*

 We have now have removed reference to Aldabergenov et al in the statistical analysis section, and as clarified in response to question 8, treatment status at time of death is determined by the National Drug Related Death Index which captures data across multiple datasets, including the national OAT treatment register (CTL):

*“We will conduct a stratified analysis for (1) deaths among people on OAT at time or death and (2) deaths among those not in treatment at time of death.”*

  

1. Discussion (cont.)

The authors, also in the discussion, imply that they can offset the challenge to establish causation with their robust methodology. This sentence needs to be corrected. The present study design does not allow proving causal relationships.

*Response:*

 It was certainly not our intention to imply our study design can offset the challenge of establishing causation or prove causal relationships and we acknowledged that this work remains hypothesis generating. We have revised this statement to remove any doubt for the reader.

*“Furthermore, while the studies employ robust methods like ITSA and comparative trend analysis of non-methadone-related deaths to explore the relationship between the intervention and observed outcomes, the study designs cannot establish causality. The Bradford Hill criteria can be used to assess causal inference; nonetheless any observed associations will remain hypothesis generating.”*

  

1. Dissemination: Regarding the dissemination in the UISCE magazine, can the authors elaborate a bit on this? Will this publication be led or co-led with people with lived/ing experience?

*Response:*

 We have an ongoing and active collaboration with UISCE for both research and educational activities. UISCE was involved in the development of this research grant, study design and the writing of the study protocol. The UISCE publication will be co-led with people with lived/living experience who are linked in with UISCE. We will organise meetings with UISCE/people who use opioids and those with experience of OAT services, and discuss how best to design/ write this piece. We will be flexible in the process of writing the magazine article, and remain open to different ideas from experts with lived experience.

 References:

 Aldabergenov, D., Reynolds, L., Scott, J., Kelleher, M., Strang, J., Copeland, C., & Kalk, N. (2022). Methadone and buprenorphine-related deaths among people prescribed and not prescribed Opioid Agonist Therapy during the COVID-19 pandemic in England. *International Journal of Drug Policy*, *110*, 103877. https://doi.org/10.1016/j.drugpo.2022.103877

 Cousins, G., Boland, F., Barry, J., Lyons, S., Keenan, E., O’Driscoll, D., Bennett, K., & Fahey, T. (2017). J-shaped relationship between supervised methadone consumption and retention in methadone maintenance treatment (MMT) in primary care: National cohort study. *Drug and Alcohol Dependence*, *173*, 126–131. https://doi.org/10.1016/j.drugalcdep.2016.12.009

 Delargy, I., Crowley, D., & Van Hout, M. C. (2019). Twenty years of the methadone treatment protocol in Ireland: Reflections on the role of general practice. *Harm Reduction Journal*, *16*(1), 5. https://doi.org/10.1186/s12954-018-0272-4

 Durand, L., Boland, F., Harnedy, N., Delargy, Í., Scully, M., Bourke, M., Ebbitt, W., Otero Vázquez, M., Keenan, E., & Cousins, G. (2023). Impact of changes to the delivery of opioid agonist treatment, introduced during the COVID-19 pandemic, on treatment access and dropout in Ireland: An interrupted time series analysis. *Journal of Substance Use and Addiction Treatment*, *149*, 209029. https://doi.org/10.1016/j.josat.2023.209029

 Durand, L., Boland, F., O’Driscoll, D., Bennett, K., Barry, J., Keenan, E., Fahey, T., & Cousins, G. (2021). Factors associated with early and later dropout from methadone maintenance treatment in specialist addiction clinics: A six-year cohort study using proportional hazards frailty models for recurrent treatment episodes. *Drug and Alcohol Dependence*, *219*, 108466. https://doi.org/10.1016/j.drugalcdep.2020.108466

 Durand, L., O’Driscoll, D., Boland, F., Keenan, E., Ryan, B. K., Barry, J., Bennett, K., Fahey, T., & Cousins, G. (2020). Do interruptions to the continuity of methadone maintenance treatment in specialist addiction settings increase the risk of drug‐related poisoning deaths? A retrospective cohort study. *Addiction*, *115*(10), 1867–1877. https://doi.org/10.1111/add.15004

 EMCDDA. (2012). *Treatment demand indicator (TDI) standard protocol 3.0: Guidelines for reporting data on people entering drug treatment in European countries*. https://www.emcdda.europa.eu/publications/manuals/tdi-protocol-3.0_en

 Gomes, T., Campbell, T. J., Kitchen, S. A., Garg, R., Bozinoff, N., Men, S., Tadrous, M., Munro, C., Antoniou, T., Werb, D., & Wyman, J. (2022). Association Between Increased Dispensing of Opioid Agonist Therapy Take-Home Doses and Opioid Overdose and Treatment Interruption and Discontinuation. *JAMA*, *327*(9), 846–855. https://doi.org/10.1001/jama.2022.1271

 Hanrahan, M. T., Millar, S., Phillips, K. P., Reed, T. E., Mongan, D., & Perry, I. J. (2022). *Problematic opioid use in Ireland, 2015–2019.* [Report]. Health Research Board. https://www.drugsandalcohol.ie/35856/

 Kelleher, C., Carew, A. M., & Lyons, S. (2021). *National Drug Treatment Reporting System 2014 – 2020 drug treatment data.* [Report]. Health Research Board. https://www.drugsandalcohol.ie/34162/

# Reviewer report: Reviewer response for version 1

**DOI:** 10.21956/hrbopenres.15418.r46011

**Published:** 2025-03-05

**Authors:** Kristen A Morin

**Affiliations:** 1 Northern Ontario School of Medicine University, Sudbury, Ontario, Canada

Thank you for the opportunity to review this protocol paper on changes in prescribing patterns after take-home dose policy changes. This is a very important topic and a very well designed study. I have a few specific suggestions below.

 INTRODUCTION

 I really like how the first paragraph is framed

 I think there’s a paragraph missing before the last paragraph. This is usually the “hook” the so what and why the research is needed after summarizing the literature and the rationale. Please refer to this article for more details. [Ref 1]

 METHODS

 Add a section named “setting” to describe the OAT climate in Ireland for the international reader. Describe the drug supply, how OAT is typically dispensed (i.e. primary care or specialty clinics like in North America) and differences in patients between both settings. Also describe the health care system in Ireland briefly (i.e. private like US or public like Canada; talk about coverage for prescription drugs like methadone and buprenorphine, etc.)

 In study 2, Can authors describe if they are only looking at one treatment window (i.e. first window as many other have done in previous studies) or considering all windows in a repeated measures model. If only looking at one window, are they adjusting for multiple treatment attempts.

 Can authors describe how or if they addressed switching between methadone and suboxone within treatment windows.

 Limitations

 The limitation that only 40% of OAT patients (primary care patients that are more stable) should be stated earlier in the methods in the “setting” section suggested above.

Is the study design appropriate for the research question?

Yes

Is the rationale for, and objectives of, the study clearly described?

Yes

Are sufficient details of the methods provided to allow replication by others?

Partly

Are the datasets clearly presented in a useable and accessible format?

Not applicable

Reviewer Expertise:

Opioid agonist treament (OAT), epidemiology, health services research

I confirm that I have read this submission and believe that I have an appropriate level of expertise to confirm that it is of an acceptable scientific standard, however I have significant reservations, as outlined above.

## References

[1] : Joining a conversation: the problem/gap/hook heuristic. *Perspect Med Educ* .2015;4(5) : 10.1007/s40037-015-0211-y 252-253 10.1007/s40037-015-0211-y 26346497 PMC4602011

# Response

**Published:** 2025-04-04

**Authors:** Louise Durand

**Affiliations:** Royal College of Surgeons in Ireland, Ireland

**INTRODUCTION:** I really like how the first paragraph is framed. I think there’s a paragraph missing before the last paragraph. This is usually the “hook” the so what and why the research is needed after summarizing the literature and the rationale. Please refer to this article for more details. [Ref 1]

***Response: *** Thank you for this useful reference and for the suggestion. We have added a paragraph introducing more explicitly the need for the proposed studies:

 “ *Although guidance regarding OAT take-home dosing changed in Ireland in 2020, there is no evidence published on the actual changes in prescribing practices of take-home dosing in Ireland, and whether any such changes were sustained over time. In addition, it is important to assess any potential impacts of changes to take-home dosing on patient outcomes, including treatment discontinuation, a known risk factor for mortality, and overdose deaths. We will address these questions through three interlinked objectives”*

**METHODS:** Add a section named “setting” to describe the OAT climate in Ireland for the international reader. Describe the drug supply, how OAT is typically dispensed (i.e. primary care or specialty clinics like in North America) and differences in patients between both settings. Also describe the health care system in Ireland briefly (i.e. private like US or public like Canada; talk about coverage for prescription drugs like methadone and buprenorphine, etc.)

***Response: *** Thank you for this suggestion. We included a setting section which provides a detailed description of the delivery of OAT in Ireland:

*“* *Methadone and buprenorphine are available free of charge to all persons undergoing OAT for opioid use disorder in Ireland. In 1998 the Misuse of Drugs (Supervision of Prescription and Supply of Methadone) Regulations were introduced in Ireland, which involved the establishment of a national register, the Central Treatment List (CTL). The Misuse of Drugs Regulations were updated in 2017 to authorise access to buprenorphine or buprenorphine/naloxone for OAT on the same statutory basis as methadone. All individuals in receipt of OAT are registered on the CTL, with each person linked to one specific prescriber and a single pharmacy dispensing site. A total of 10,251 people were in receipt of OAT in 2019* *(Durand et al., 2023)* *. OAT is provided in specialist outpatient addiction clinics or primary care settings, with approximately 60% of people in treatment in specialist addiction clinics* *(Delargy et al., 2019; Durand et al., 2021)* *. Previous studies of OAT in Ireland suggest that access to take-home doses is greater in primary care than in outpatient clinics* *(Cousins et al., 2017; Durand et al., 2020)* *.* *”*

 In study 2, Can authors describe if they are only looking at one treatment window (i.e. first window as many other have done in previous studies) or considering all windows in a repeated measures model. If only looking at one window, are they adjusting for multiple treatment attempts.

***Response: *** Consistent with previous studies (Gomes et al., 2022), we plan to look at one exposure window and whether THD regimen changed during this time. We are not planning to include further treatment episodes after the first dropout in the analysis, as we consider further episodes may not be comparable to those ongoing at the time of the guidelines introduction, due to variations in public health restrictions.

 Can authors describe how or if they addressed switching between methadone and suboxone within treatment windows.

***Response: *** Thank you for highlighting this point. Most people receive methadone in Ireland, with low numbers receiving buprenorphine. While we do anticipate a small increase in buprenorphine, the numbers are likely to be very small. This is based on our previous analysis of the national treatment register, where we identified that 2% of the 10,251 people on OAT were prescribed buprenorphine (n=178) in March 2020 (Durand et al., 2023). We would be underpowered to examine switching. We plan to exclude patients who switched between treatments from the main analysis. However, we will report on the numbers to describe the level of switching:

*” People who had their OAT drug changed (from methadone to buprenorphine or vice versa) during the accrual or exposure windows will be excluded.”*

 Limitations: The limitation that only 40% of OAT patients (primary care patients that are more stable) should be stated earlier in the methods in the “setting” section suggested above.

***Response: *** Thank you for this suggestion, we have included this detail in the setting section as you recommended.

*“* *Methadone and buprenorphine are available free of charge to all persons undergoing OAT for opioid use disorder in Ireland. In 1998 the Misuse of Drugs (Supervision of Prescription and Supply of Methadone) Regulations were introduced in Ireland, which involved the establishment of a national register, the Central Treatment List (CTL). The Misuse of Drugs Regulations were updated in 2017 to authorise access to buprenorphine or buprenorphine/naloxone for OAT on the same statutory basis as methadone. All individuals in receipt of OAT are registered on the CTL, with each person linked to one specific prescriber and a single pharmacy dispensing site. A total of 10,251 people were in receipt of OAT in 2019 *(Durand et al., 2023) *. OAT is provided in specialist outpatient addiction clinics or primary care settings, with approximately 60% of people in treatment in specialist addiction clinics *(Delargy et al., 2019; Durand et al., 2021) *. Previous studies of OAT in Ireland suggest that access to take-home doses is greater in primary care than in outpatient clinics *(Cousins et al., 2017; Durand et al., 2020) *.* *”*

**References:**

 Cousins, G., Boland, F., Barry, J., Lyons, S., Keenan, E., O’Driscoll, D., Bennett, K., & Fahey, T. (2017). J-shaped relationship between supervised methadone consumption and retention in methadone maintenance treatment (MMT) in primary care: National cohort study. *Drug and Alcohol Dependence*, *173*, 126–131. https://doi.org/10.1016/j.drugalcdep.2016.12.009

 Delargy, I., Crowley, D., & Van Hout, M. C. (2019). Twenty years of the methadone treatment protocol in Ireland: Reflections on the role of general practice. *Harm Reduction Journal*, *16*(1), 5. https://doi.org/10.1186/s12954-018-0272-4

 Durand, L., Boland, F., Harnedy, N., Delargy, Í., Scully, M., Bourke, M., Ebbitt, W., Otero Vázquez, M., Keenan, E., & Cousins, G. (2023). Impact of changes to the delivery of opioid agonist treatment, introduced during the COVID-19 pandemic, on treatment access and dropout in Ireland: An interrupted time series analysis. *Journal of Substance Use and Addiction Treatment*, *149*, 209029. https://doi.org/10.1016/j.josat.2023.209029

 Durand, L., Boland, F., O’Driscoll, D., Bennett, K., Barry, J., Keenan, E., Fahey, T., & Cousins, G. (2021). Factors associated with early and later dropout from methadone maintenance treatment in specialist addiction clinics: A six-year cohort study using proportional hazards frailty models for recurrent treatment episodes. *Drug and Alcohol Dependence*, *219*, 108466. https://doi.org/10.1016/j.drugalcdep.2020.108466

 Durand, L., O’Driscoll, D., Boland, F., Keenan, E., Ryan, B. K., Barry, J., Bennett, K., Fahey, T., & Cousins, G. (2020). Do interruptions to the continuity of methadone maintenance treatment in specialist addiction settings increase the risk of drug‐related poisoning deaths? A retrospective cohort study. *Addiction*, *115*(10), 1867–1877. https://doi.org/10.1111/add.15004
